# Closed-form solution of oscillating Maxwell nano-fluid with heat and mass transfer

**DOI:** 10.1038/s41598-022-16503-w

**Published:** 2022-07-16

**Authors:** Aamir Farooq, Sadique Rehman, Abdulaziz N. Alharbi, Muhammad Kamran, Thongchai Botmart, Ilyas Khan

**Affiliations:** 1 Department of Mathematics, Zhejiang Normal University, Jinhua, 321004 Zhejiang China; 2 Department of Mathematics, Abbottabad University of Science and Technology, Abbottabad, Pakistan; 3 Department of Pure and Applied Mathematics, University of Haripur, Haripur, KPK Pakistan; 4 Department of Physics, College of Science, Taif University, P.O.Pox 11099, Taif, 21944 Saudi Arabia; 5 Department of Mathematics, COMSATS University Islamabad, Wah Campus, Islamabad, 47040 Pakistan; 6 Department of Mathematics, Faculty of Science, Khon Kaen University, Khon Kaen, 40002 Thailand; 7 Department of Mathematics, College of Science Al-Zulfi, Majmaah University, Al-Majmaah, 11952 Saudi Arabia

**Keywords:** Engineering, Mathematics and computing, Nanoscience and technology

## Abstract

The primary goal of this article is to analyze the oscillating behavior of Maxwell Nano-fluid with regard to heat and mass transfer. Due to high thermal conductivity of engine oil is taken as a base fluid and graphene Nano-particles are introduced in it. Coupled partial differential equations are used to model the governing equations. To evaluate the given differential equations, certain dimensionless factors and Laplace transformations are used. The analytical solution is obtained for temperature, concentration and velocity. The temperature and concentration gradient are also finds to analyze the rate of heat and mass transfer. As a special case, the solution for Newtonian fluid is discussed. Finally, the behaviors of various physical factors are studied graphically for both sine and cosine oscillation and give physical meanings to the parameters.

## Introduction

A large number of scientists and engineers are keen in knowing the computational and anatomical features of industrial fluid in recent times, because of their increasing usage in engineering and industrial sciences. Such fluids are categorized as non-Newtonian fluid, and their sub-division contains cement, drilling mud, synthetic oils, asphalts and many more^1^. Some of the fluids show both the viscous and elastic properties such fluid are called viscoelastic fluids like toothpaste, polymers solutions, paints, clay etc.^2^. The three major types of non-Newtonian fluids are Integral form, rate and differential type fluids. The Maxwell fluid is the principal viscoelastic rate type liquid, which is likewise broadly used. The differential structure and rate type models have stood out enough to be noticed among them. Due to the straightforwardness of rate type liquid, numerous examiners are giving specific consideration to Maxwell fluid^3–6^. Riaz et al.^7,8^ studied the Maxwell fluid of heat and mass transport in term of local and non-local differential operators. The semi analytical solution was obtained via Laplace transform. A fractional Maxwell fluid was analyzed by^9,10^ using numerical techniques. Natural convection flow of Maxwell fluid between vertical plates was investigated by Na,W et al.^11^. A closed- form solution was acquired through Laplace transform. Khan et al.^12,13^ studied mixed convection Maxwell fluid ordinary and fractionally over oscillating vertical plate. Exact solution and some special cases for Newtonian fluid was obtained through Laplace transform. Abro, K. A. et al.^14^ also obtained analytical solution of Maxwell fluid over vertical plane. Numerical solution of comparative Maxwell and Casson fluid was illustrated by Kumar et al.^15^ using Runge- Kutta based shooting method. Sodium alginate (SA-NaAlg) based (MoS2) nanofluid was researched by Ahmed et al.^16^ utilizing Maxwell Garnetts and Brinkman models. Physically, mixed convection is induced because of upgrade force and abrupt plate motion. Farooq et al.^17^ analyzed the generalized Maxwell model flow of magnetic hydrodynamic (MHD) fluid through porous duct. The solution was obtained via double Fourier sine and Laplace transform. Exact solutions for unsteady MHD flow of Maxwell fluid over oscillating plate have been illustrated by^18,19^. Sandeep et al.^20^ discussed the comparative study of Jeffery, Maxwell and Oldroyd-B fluid through extended surface utilizing similarity transformation and solution was acquired numerically via Runge–Kutta dependent shooting method. A fractional Maxwell model in porous medium was illustrated by Aman, S et al.^21^. The numerical solution was obtained using Stehfest’s algorithm. Fetecau et al.^22^ studied the second problem of stokes for Maxwell fluid via Laplace transform.

Coupled heat and mass transport is a process that happens commonly in nature. It exists not only as a result of temperature variations, but also because of concentration variations or the combine effect of these two. The impact of a compound response is dictated by, whether it is homogeneous or heterogeneous. The incorporation of unadulterated water and air, are inconceivable in nature. It's conceivable that any external matter is normally there, or that it's blended in with air or water. At the point when an external mass is available in air or fluids, it prompts a synthetic response. Numerous substance advances, like the assembling of pottery, the creation of polymers and food handling, benefits from the investigation of related synthetic responses. Shateyi^23^ considered the Maxwell fluid on an extended sheet over Darcian medium. The general solution for natural convection flow past on a vertical plate with heat and mass transfer was discussed by^24,25^. Free convection flow with heat and mass transfer over fluctuating and accelerated vertical plate through porous medium was studied by^26–28^. Closed-form solution was obtained via Laplace transform method. Rajput et al.^29^ researched the impact of radiation on an impulsively vertical plate with heat and mass transmission of MHD flow. Pattnaik, J. R et al.^30^ addressed the MHD flow over exponentially inclined plate via porous medium. For solving the given equations Laplace transformation was utilized. Seth et al.^31^ illustrated the MHD convected flow of Soret and Hall effects in a rotating system with heat and mass transmission. Kumam, P et al.^32^ explained the comparative study of fractional Maxwell fluid. Semi analytical solution was obtained via Laplace transformation. Fetecau et al.^33^ examine the impact of radiation and porosity on MHD fluid on an oscillating vertical layer. Tang et al.^34^ have given the comparison of two different fractional definitions (Caputo, Caputo–Fabrizio) of Maxwell fluid.

Nanofluids are being used to improve the thermal conductivity of base fluids such as water, engine oil, propylene glycol, and ethylene glycol, among others. They have a wide range of uses in engineering and biomedicine including cooling, cancer treatment and industrial plants. The use of solid particles suspension to improve the thermal conductivity of traditional heat transfer fluid is a relatively new advancement in engineering technology. This technology has been recently paired with advance nanofluids and liquid nanoparticles suspensions technologies, to establish a new group of nanofluids based on solar collectors. Aman et al.^35^ studied the natural convection flow of Maxwell fluid with graphene nanoparticles. Murtaza et al.^36^ examined the concrete nanoparticles in fractional Maxwell fluid. Exact solution was acquired via LT. The Maxwell hybrid nano-fluid of convective flow in a channel was discussed by^37,38^. Asjad et al.^39^ investigated the clay-nanoparticles of generalized Maxwell fluid in heat transmission via infinite flat surface. Wang et al.^40^ argued the Oldroyd-B nanofluid of MHD natural convection flow via permeable medium. Arif et al.^41^ studied the Maxwell hybrid nanofluid (engine oil) in vibratory vertical cylinder. Kumar et al.^42,45^ studied the impact of magnetic dipole on thermophoretic particle deposition in the flow of Maxwell fluid and nanofluid over a stretching sheet. Prasannakumara^43^ focused on the nuumerical simulation of heat transport in Maxwell nanofluid flow over a stretching sheet considering magnetic dipole effect. Through the stretchable disks slip flow of Casson–Maxwell nanofluid was studied by Gowda et al.^44^. Also, many other authors focused to studied Maxwell nanofluid, see e.g.^46–48^ for better understanding. Cheng et al.^50^ proposed the heat transfer analysis of elastoviscoplastic non-Newtonian generalized fluid with hybrid nanofluid and dust particles. Numerical solution of the model is acquired via shooting method. Kaneez et al.^51,52^ investigated the numerical solution of micropolar fluid including dusty, mono and hybrid nono-structures. Khan et al.^53^ demonstrated the upper convected Maxwell MHD micropolar fluid with the impact of Joule heating and thermal radiation utilizing a hyperbolic heat flux.

To the best of author’s knowledge no one has consider the oscillating Maxwell nanofluid with the heat and mass transfer. So, motivated by this we study this problem analytically. The aim of this work is to explore oscillating Maxwell nanofluid with heat and mass transfer. The suspension of graphene nanoparticles and engine oil (base fluid) is taken in consideration. The governing equation is solved through LT. The solution for temperature, concentration and velocity are calculated analytically. Temperature slope and concentration gradient in the form of Nusslet number and Sherwood number are also acquired. Finally, the influence of various embedded factors on temperature, concentration and velocity shows graphically as well as theoretically.

## Problem statement

**Figure 1:**
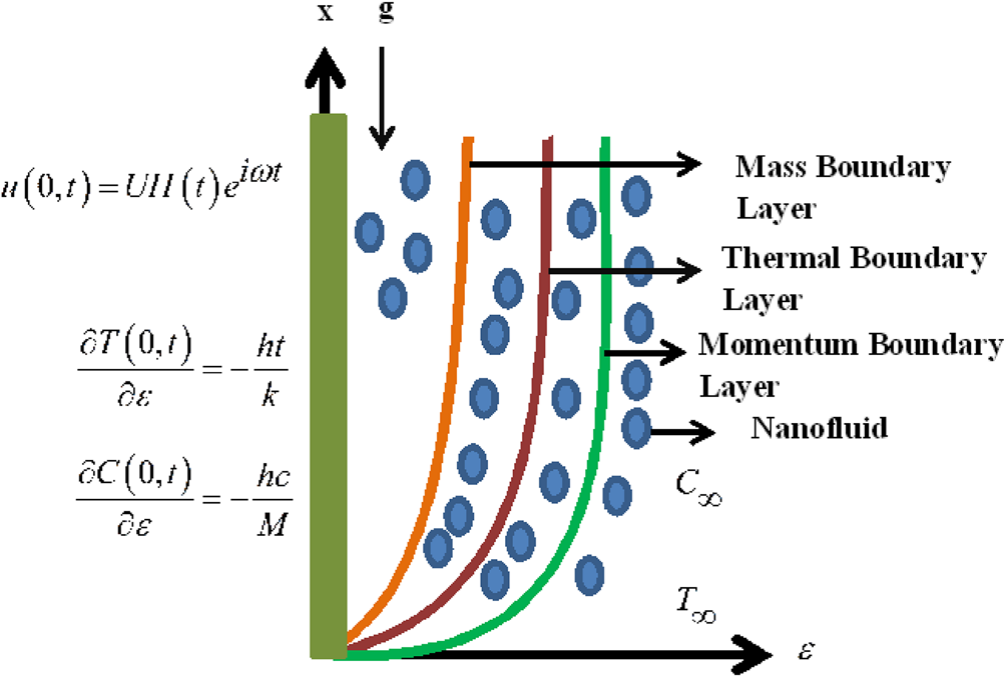
Sketch of the flow model.

Let us assume Maxwell nanofluid passed on an infinite oscillating vertical plate with heat and mass transfer. $\varepsilon$ is perpendicular to the plate while plate along *x*-axis. Both the fluid and the plate are initially at rest with ambient temperature ${T}_{\infty }$ and ambient concentration ${C}_{\infty }$. After some time at $t={0}^{+}$ the plate begins oscillation in its plane ($\varepsilon =0$) as indicated with velocity $\underline {U} H\left( t \right)e^{{\left( {i\omega t} \right)i}}$, where $\underline {U}$ is the amplitude, $\omega$ represents the frequency of the oscillation of the plate, $H\left( t \right)$ is the unit step function and $i$ is the unit vector in the vertical flow direction. Chemical reaction phenomenon is also incorporated to elaborate mass diffusion response. We suppose that the velocity, concentration and velocity is the function of $\varepsilon \text{ and }t.$ The governing equations is model in the following form. Figure 1 shows the geometry of the flow problem.

1 $${\rho }_{nf}\left(1+{\lambda }_{0}\frac{\partial }{\partial t}\right)\frac{\partial u\left(\varepsilon ,t\right)}{\partial t}={\mu }_{nf}\frac{{\partial }^{2}u\left(\varepsilon ,t\right)}{\partial {\varepsilon }^{2}}+\left(1+{\lambda }_{0}\frac{\partial }{\partial t}\right)g{\left(\rho {\beta }_{t}\right)}_{nf}\left(T-{T}_{\infty }\right)+\left(1+{\lambda }_{0}\frac{\partial }{\partial t}\right)g{\left(\rho {\beta }_{m}\right)}_{nf}\left(C-{C}_{\infty }\right)$$

2 $$\left( {\rho c_{p} } \right)_{nf} \frac{\partial T(\varepsilon ,t)}{{\partial t}} = k_{nf} \frac{{\partial^{2} T(\varepsilon ,t)}}{{\partial \varepsilon^{2} }} - \frac{{\partial q_{r} }}{\partial \varepsilon }$$

3 $$\frac{\partial C(\varepsilon ,t)}{\partial t}=M\frac{{\partial }^{2}C(\varepsilon ,t)}{\partial {\varepsilon }^{2}}-K\left(C-{C}_{\infty }\right)$$

Here, $\rho_{nf}$ is the density of nanofluid, $\lambda_{o}$ represents the Maxwell fluid parameter, $\mu_{nf}$ denotes the dynamic viscosity of nanofluid, $g$ is the gravitational acceleration, $\left( {\beta_{t} } \right)_{nf}$ represents the coefficient of thermal expansion of nanofluid, $c_{p}$ denotes the specific heat, $\left( {\beta_{m} } \right)_{nf}$ represents the coefficient of mass expansion of nanofluid, $K$ represents the chemical reaction parameter, $M_{nf}$ shows the diffusion species coefficient.

The corresponding initial conditions (ICs) and boundary conditions (BCs) are of the following form^49^:

4 $$\begin{array}{*{20}c} {u\left( {\varepsilon ,0} \right) = 0,T\left( {\varepsilon ,0} \right) = T_{\infty } ,C\left( {\varepsilon ,0} \right) = C_{\infty } ,} \\ {u\left( {0,t} \right) = \underline {U} H\left( t \right)e^{i\omega t} ,\frac{{\partial T\left( {0,t} \right)}}{\partial \varepsilon } = - \frac{ht}{k},\frac{{\partial C\left( {0,t} \right)}}{\partial \varepsilon } = - \frac{hc}{M},\varepsilon ,t > 0} \\ {u\left( {\varepsilon ,t} \right) = 0,T\left( {\varepsilon ,t} \right) = T_{\infty } ,C\left( {\varepsilon ,t} \right) = C_{\infty } as \varepsilon \to \infty . } \\ \end{array}$$

where $T_{\infty }$ is the ambient temperature, $ht$ represents the coefficient of heat transfer, $C_{\infty }$ demonstrates the ambient concentration, and $hc$ shows the coefficient of mass transfer.

Using Rosseland approximations^9,31,33^ and gaining the small temperature variation between the temperature ${T}_{\infty }$ of the free stream and the fluids temperature $T,$ exploring the Taylor theorem on ${T}^{4}$ about ${T}_{\infty }$ and omitting the numbers of 2nd and higher order, we get

5 $${q}_{r}=-\frac{4{\phi }^{*}}{3{\kappa }^{*}}\frac{\partial {T}^{4}}{\partial \varepsilon }$$

and

6 $$T^{4} \cong 4T_{\infty }^{3} T - 3T_{\infty }^{4}$$

where $\phi^{*} ,\kappa^{*}$ are respectively Stefan boltzman constants, is the mean absorption coefficient.

Substituting (5) into (2) we obtain the following form

7 $$\left( {\rho c_{p} } \right)_{nf} \frac{\partial T(\varepsilon ,t)}{{\partial t}} = k_{nf} \frac{{\partial^{2} T(\varepsilon ,t)}}{{\partial \varepsilon^{2} }} + \frac{{16\phi^{*} T_{\infty }^{3} }}{{3\kappa^{*} }}\frac{{\partial^{2} T(\varepsilon ,t)}}{{\partial \varepsilon^{2} }}$$

The thermo-physical characteristics of nanoparticles were given by^35^;

$$\begin{gathered} \rho_{nf} = \left( {1 - \vartheta } \right)\rho_{f} + \vartheta \rho_{s} , \, \mu_{nf} { = }\left( {1 - \vartheta } \right)^{ - 2.5} \mu_{f} , \, \left( {\rho \beta_{t} } \right)_{nf} = \left( {1 - \vartheta } \right)\left( {\rho \beta_{t} } \right)_{f} + \vartheta \left( {\rho \beta_{t} } \right)_{s} , \hfill \\ \left( {\rho \beta_{m} } \right)_{nf} = \left( {1 - \vartheta } \right)\left( {\rho \beta_{m} } \right)_{f} + \vartheta \left( {\rho \beta_{m} } \right)_{s} , \, \left( {\rho c_{p} } \right)_{nf} = \left( {1 - \vartheta } \right)\left( {\rho c_{p} } \right)_{f} + \vartheta \left( {\rho c_{p} } \right)_{s} , \hfill \\ \frac{{k_{nf} }}{{k_{f} }} = \frac{{\left( {k_{s} + 2k_{f} } \right) - 2\vartheta \left( {k_{f} - k_{s} } \right)}}{{\left( {k_{s} - 2k_{f} } \right) + \vartheta \left( {k_{f} - k_{s} } \right)}}. \hfill \\ \end{gathered}$$

The dimensionless parameters are given below;

8 $$\mathop u\limits^{^\circ } = \frac{u}{{\underline{U} }}, \, \mathop t\limits^{^\circ } = \frac{{\underline{U}^{2} }}{{\underline{\upsilon } }}t, \, \mathop \omega \limits^{^\circ } = \frac{{\underline{\upsilon } }}{{\underline{U}^{2} }}\omega , \, \mathop y\limits^{^\circ } = \frac{{\underline{U} }}{{\underline{\upsilon } }}y, \, \mathop \lambda \limits^{^\circ } = \frac{{\underline{U}^{2} }}{{\underline{\upsilon } }}\lambda_{0}$$

After substituting the above dimensionless parameters in Eqs. (1), (3) and (7) we get these governing dimensionless equations and dropping the $^\circ$ from the above dimensionless factors,

9 $$\vartheta_{1} \left( {1 + \lambda \frac{\partial }{\partial t}} \right)\frac{\partial u(\varepsilon ,t)}{{\partial t}} = \vartheta_{2} \frac{{\partial u^{2} (\varepsilon ,t)}}{{\partial \varepsilon^{2} }} + \left( {1 + \lambda \frac{\partial }{\partial t}} \right)\vartheta_{3} Gr_{t} \theta (\varepsilon ,t) + \left( {1 + \lambda \frac{\partial }{\partial t}} \right)\vartheta_{4} Gr_{m} \hat{C}(\varepsilon ,t),$$

10 $$\vartheta_{5} \Pr \frac{\partial \theta (\varepsilon ,t)}{{\partial t}} = \vartheta_{6} \left( {1 + Rd} \right)\frac{{\partial^{2} \theta (\varepsilon ,t)}}{{\partial \varepsilon^{2} }},$$

11 $$Sc\frac{\partial C(\varepsilon ,t)}{{\partial t}} = \frac{{\partial^{2} C(\varepsilon ,t)}}{{\partial \varepsilon^{2} }} - ScKC(\varepsilon ,t)$$

where

$$\begin{gathered} \theta = \frac{{\underline{U} k}}{{\underline{\upsilon } ht}}\left( {T - T_{\infty } } \right),{\text{ Pr}} = \frac{{\mu c_{p} }}{k}, \, Gr_{t} = \left( {\frac{{\underline{\upsilon } }}{{\underline{U}^{2} }}} \right)^{2} \frac{{g\beta_{t} ht}}{k}, \, Gr_{m} = \left( {\frac{{\underline{\upsilon } }}{{\underline{U}^{2} }}} \right)^{2} \frac{{g\beta_{m} hc}}{M},Sc = \frac{{\underline {\upsilon } }}{M}, \hfill \\ Rd = \frac{{16\phi^{ * } T_{\infty }^{3} }}{{3k\kappa^{ * } }}, \, \mathop K\limits^{\Lambda } = \frac{{\underline{\upsilon } K}}{{\underline{U}^{2} }}, \, \mathop C\limits^{\Lambda } = \frac{{\underline{U} M}}{{\underline{\upsilon } hc}}\left( {C - C_{\infty } } \right), \, \vartheta_{1} = \left( {1 - \vartheta } \right) + \vartheta \frac{{\rho_{s} }}{{\rho_{f} }}, \, \vartheta_{2} = \left( {1 - \vartheta } \right)^{ - 2.5} \hfill \\ \vartheta_{3} = \left( {1 - \vartheta } \right) + \vartheta \frac{{\left( {\beta_{t} \rho } \right)_{s} }}{{\left( {\beta_{t} \rho } \right)_{f} }}, \, \vartheta_{4} = \left( {1 - \vartheta } \right) + \vartheta \frac{{\left( {\beta_{m} \rho } \right)_{s} }}{{\left( {\beta_{m} \rho } \right)_{f} }}, \, \vartheta_{5} = \left( {1 - \vartheta } \right) + \vartheta \frac{{\left( {c_{p} \rho } \right)_{s} }}{{\left( {c_{p} \rho } \right)_{f} }}, \, \vartheta_{6} = \frac{{k_{nf} }}{{k_{f} }}. \hfill \\ \end{gathered}$$

Here, $\theta$ denotes the dimensionless temperature, $\Pr$ shows Prandtl number, $Gr_{t}$ demonstrates thermal Grashof number, $Gr_{m}$ represents mass Grashof number, $\vartheta$ denotes volume fraction parameter and $Sc$ represents Schmidt number.

The dimensionless ICs and BCs are as follow and skip $^\circ$ from the non-dimensional factors

12 $$\begin{gathered} u(\varepsilon ,0) = 0, \, \theta (\varepsilon ,0) = 0, \, C(\varepsilon ,0) = 0 \, \hfill \\ u(0,t) = H(t)e^{i\omega t} , \, \frac{\partial \theta (0,t)}{{\partial \varepsilon }} = - 1, \, \frac{\partial C(0,t)}{{\partial \varepsilon }} = - 1, \hfill \\ u(\varepsilon ,t) = 0, \, \theta (\varepsilon ,t) = 0, \, C(\varepsilon ,t) = 0, \, \varepsilon \to \infty . \hfill \\ \end{gathered}$$

The thermo-physical property of graphene (nanoparticles) and engine oil (base fluid) are tabulated in table. 1

**Table 1:** Properties of nano particles.

Model	$$\rho \left( {{\raise0.7ex\hbox{${{\text{kg}}}$} \!\mathord{\left/ {\vphantom {{{\text{kg}}} {{\text{m}}^{3} }}}\right.\kern-\nulldelimiterspace} \!\lower0.7ex\hbox{${{\text{m}}^{3} }$}}} \right)$$	$$c_{p} \left( {{\raise0.7ex\hbox{${\text{k}}$} \!\mathord{\left/ {\vphantom {{\text{k}} {{\text{kg}}}}}\right.\kern-\nulldelimiterspace} \!\lower0.7ex\hbox{${{\text{kg}}}$}}} \right)$$	$$k\left( {{\text{Wm}}^{ - 1} {\text{k}}^{ - 1} } \right)$$	$$\beta \times 10^{5} \,\,{\text{k}}^{ - 1}$$
Graphene	2250	2100	2500	21
Engine oil	884	1910	0.144	70

## Problem solution

### Temperature

Taking LT on Eq. (10) and also using the related ICs and BCs, we get the following transform form;

13 $$\overline{\theta } \left( {\varepsilon ,r} \right) = \frac{1}{{\sqrt {a_{0} \Pr_{eff} } r^{\frac{3}{2}} }}e^{{ - \varepsilon \sqrt {a_{0} \Pr_{eff} .r} }} ,$$

The inverse LT of Eq. (13) has the following final form,

14 $$\theta \left( {\varepsilon ,t} \right) = \frac{2\sqrt t }{{\sqrt {a_{0} \Pr_{eff} } }}\left[ {\frac{1}{\sqrt \pi }e^{{ - \varepsilon \frac{{a_{0} \Pr_{eff} }}{4t}}} - \varepsilon \frac{{\sqrt {a_{0} \Pr_{eff} } }}{2\sqrt t }erfc\left( {\varepsilon \frac{{\sqrt {a_{0} \Pr_{eff} } }}{2\sqrt t }} \right)} \right].$$

### Nusslet number

The Nusslet number, measure the rate of heat transfer at the plate can be acquired by differentiating Eq. (13) with respect to $\varepsilon$ and using $\varepsilon = 0$, we get the constant term. i.e.

15 $$Nu(t) = 1$$

This shows the heat is transfer due to purely conduction.

### Concentration

Applying LT on Eq. (11) and also utilizing the respective ICs and BCs, we acquire the following transform form;

16 $$\overline{C} \left( {\varepsilon ,r} \right) = \frac{1}{{r\sqrt {Sc\left( {K + r} \right)} }}e^{{ - \varepsilon \sqrt {Sc\left( {K + r} \right)} }}$$

The Laplace inverse transform of Eq. (16) as;

17 $$C\left( {\varepsilon ,t} \right) = \frac{1}{{2\sqrt {ScK} }}\left[ {e^{{ - \varepsilon \sqrt {ScK} }} erfc\left( {\frac{{\varepsilon \sqrt {Sc} }}{2\sqrt t } - \sqrt {Kt} } \right) - e^{{ - \varepsilon \sqrt {ScK} }} erfc\left( {\frac{{\varepsilon \sqrt {Sc} }}{2\sqrt t } + \sqrt {Kt} } \right)} \right]$$

### Sherwood number

The Sherwood number measure the mass transfer at the plate. The Sherwood number defined and represented by,

18 $$Sh = - \left( {\frac{\partial C(\varepsilon ,t)}{{\partial \varepsilon }}} \right)_{\varepsilon = 0}$$

Now to obtain Sherwood number we differentiate Eq. (16) with respect to $\varepsilon$ and utilizing $\varepsilon = 0$, we get

19 $$Sh(t) = 1$$

### Velocity

The Laplace transform of Eq. (9) and also the appropriate ICs and BCs in Eq. (12) we get the following transform form;

20 $$\begin{gathered} \overline{u} \left( {\varepsilon ,r} \right) = \left[ {\frac{1}{r - i\omega } - \frac{{a_{2} Gr_{t} }}{{\sqrt {a_{0} \Pr_{eff} } }}W_{0} (r) + \frac{{a_{3} Gr_{m} }}{{\sqrt {Sc} }}W_{11} (r)W_{3} (r)} \right] * W_{4} (\varepsilon ,r) \hfill \\ \, + \left[ {\frac{{a_{2} Gr_{t} }}{{\sqrt {a_{0} \Pr_{eff} } }}W_{0} (r)} \right] * W_{5} (\varepsilon ,r) - \left[ {\frac{{a_{3} Gr_{m} }}{{\sqrt {Sc} }}W_{3} (r)} \right] * W_{6} (\varepsilon ,r). \hfill \\ \end{gathered}$$

Here,

21 $$W_{0} (r) = \left[ {\frac{{b_{1} - b_{0} }}{{b_{1}^{2} }}\frac{1}{{r^{\frac{3}{2}} }} + \frac{{b_{0} }}{{b_{1} }}\frac{1}{{r^{\frac{5}{2}} }}\frac{{b_{0} - b_{1} }}{{b_{1}^{2} b_{11} }}\left( {\frac{{b_{11} }}{{\sqrt r \left( {r - \left( {\sqrt {b_{11} } } \right)^{2} } \right)}}} \right)} \right]$$

22 $$W_{3} (r) = W_{1} (r) - W_{2} (r)$$

23 $$W_{1} (r) = \frac{{b_{0} }}{{b_{2} - r\left( {r + b_{4} } \right)}}$$

24 $$W_{2} (r) = \frac{r}{{b_{2} - r\left( {r + b_{4} } \right)}}$$

25 $$W_{4} (\varepsilon ,r) = e^{{ - \varepsilon \sqrt {\lambda a_{1} } \sqrt {\left( {r + \frac{{b_{0} }}{2}} \right)^{2} - \left( {\frac{{b_{0} }}{2}} \right)^{2} } }}$$

26 $$W_{51} (\varepsilon ,r) = e^{{ - \varepsilon \sqrt {a_{0} \Pr_{eff} } \sqrt r }}$$

27 $$W_{6} (\varepsilon ,r) = \frac{{e^{{ - \varepsilon \sqrt {Sc\left( {K + r} \right)} }} }}{{r\sqrt {\left( {K + r} \right)} }}$$

28 $$W_{12} (r) = W_{11} (r)W_{3} (r)$$

The inverse LT of Eqs. (21)–(27), we obtain

29 $$W_{0} (t) = \left[ {\frac{{b_{1} - b_{0} }}{{b_{1}^{2} }}2\sqrt {\frac{t}{\pi }} + \frac{{b_{0} }}{{b_{1} }}\frac{4t}{3}\sqrt {\frac{t}{\pi }} + \frac{{b_{0} - b_{1} }}{{b_{1}^{2} b_{11} }}e^{{b_{11} t}} erf\left( {\sqrt {b_{11} t} } \right)} \right]$$

30 $$W_{1} (t) = b_{0} \left[ {\frac{{e^{{t\left( {\frac{{ - b_{4} }}{2} - \frac{1}{2}\sqrt {4b_{2} + b_{4}^{2} } } \right)}} - e^{{t\left( {\frac{{ - b_{4} }}{2} + \frac{1}{2}\sqrt {4b_{2} + b_{4}^{2} } } \right)}} }}{{\sqrt {4b_{2} + b_{4}^{2} } }}} \right]$$

31 $$W_{11} (t) = \frac{{Erf\left( {\sqrt {Kt} } \right)}}{\sqrt K }$$

32 $$W_{2} (t) = \left[ {\frac{{b_{4} e^{{t\left( { - \frac{{b_{4} }}{2} - \frac{1}{2}\sqrt {4b_{2} + b_{4}^{2} } } \right)}} - b_{4} e^{{t\left( { - \frac{{b_{4} }}{2} + \frac{1}{2}\sqrt {4b_{2} + b_{4}^{2} } } \right)}} + \sqrt {4b_{2} + b_{4}^{2} } e^{{t\left( { - \frac{{b_{4} }}{2} - \frac{1}{2}\sqrt {4b_{2} + b_{4}^{2} } } \right)}} + \sqrt {4b_{2} + b_{4}^{2} } e^{{t\left( { - \frac{{b_{4} }}{2} + \frac{1}{2}\sqrt {4b_{2} + b_{4}^{2} } } \right)}} }}{{2\sqrt {4b_{2} + b_{4}^{2} } }}} \right]$$

33 $$\begin{aligned} w_{1} (\varepsilon ,t) &= \left[ {W_{5} (\varepsilon ,t) + \frac{1}{{2a_{1} \lambda }}\int\limits_{0}^{t} {W_{5} (\varepsilon ,p)\frac{p}{{\sqrt {t^{2} - p^{2} } }}I_{1} \left( {\frac{1}{{2a_{1} \lambda }}\sqrt {t^{2} - p^{2} } } \right)dp} } \right]e^{{ - \frac{1}{{2a_{1} \lambda }}t}} \hfill \\ & = \frac{\varepsilon \sqrt t }{{2t\sqrt {\pi t} }}e^{{\left( { - \frac{{\varepsilon^{2} a_{1} \lambda }}{4t} - \frac{1}{{2a_{1} \lambda }}t} \right)}} + \frac{1}{{2a_{1} \lambda }}e^{{ - \frac{1}{2\lambda }t}} \hfill \\ &\quad \int\limits_{0}^{t} {\frac{{\varepsilon \sqrt {a_{1} \lambda } }}{{2z\sqrt {\pi p} }}e^{{\left( { - \frac{{\varepsilon^{2} a_{1} \lambda }}{4p}} \right)}} \frac{p}{{\sqrt {t^{2} - p^{2} } }}} I_{1} \left( {\frac{1}{{2a_{1} \lambda }}\sqrt {t^{2} - p^{2} } } \right)dp, \hfill \\ \end{aligned}$$

34 $$W_{4} (\varepsilon ,t) = L^{ - 1} \left\{ {W_{4} (\varepsilon ,r)} \right\} = \left\{ \begin{gathered} w_{1} (\varepsilon ,t); \, \varepsilon > 0 \hfill \\ \delta (t); \, \varepsilon = 0 \hfill \\ \end{gathered} \right.$$

35 $$W_{5} (\varepsilon ,t) = L^{ - 1} \left\{ {W_{51} (\varepsilon ,r)} \right\} = \left\{ \begin{gathered} \frac{{\varepsilon \sqrt {a_{0} \Pr_{eff} } e^{{ - \varepsilon^{2} \frac{{a_{0} \Pr_{eff} }}{4t}}} }}{{2t\sqrt {\pi t} }}; \, \varepsilon { > 0} \hfill \\ \delta {\text{(t); }}\varepsilon = 0. \hfill \\ \end{gathered} \right.$$

36 $$W_{6} \left( {\varepsilon ,t} \right) = \frac{1}{2\sqrt K }\left[ {e^{{ - \varepsilon \sqrt {ScK} }} erfc\left( {\frac{{\varepsilon \sqrt {Sc} }}{2\sqrt t } - \sqrt {Kt} } \right) - e^{{ - \varepsilon \sqrt {ScK} }} erfc\left( {\frac{{\varepsilon \sqrt {Sc} }}{2\sqrt t } + \sqrt {Kt} } \right)} \right]$$

The Laplace inverse transform of Eq. (20) and utilizing the Faltung theoram, we get

37 $$\begin{aligned} u\left( {\varepsilon ,t} \right) &= \int\limits_{0}^{t} {e^{i\omega (t - v)} W_{4} \left( {\varepsilon ,v} \right)dv - \frac{{a_{2} Gr_{t} }}{{\sqrt {a_{0} \Pr_{eff} } }}\int\limits_{0}^{t} {W_{0} (t - v)} } W_{4} \left( {\varepsilon ,v} \right)dv + \frac{{a_{3} Gr_{m} }}{{\sqrt {Sc} }}\int\limits_{0}^{t} {W_{12} (t - v)W_{4} \left( {\varepsilon ,v} \right)dv} \hfill \\ &\quad + \frac{{a_{2} Gr_{t} }}{{\sqrt {a_{0} \Pr_{eff} } }}\int\limits_{0}^{t} {W_{0} (t - v)} W_{5} \left( {\varepsilon ,v} \right)dv - \frac{{a_{3} Gr_{m} }}{{\sqrt {Sc} }}\int\limits_{0}^{t} {W_{3} (t - v)} W_{6} \left( {\varepsilon ,v} \right)dv \hfill \\ \end{aligned}$$

## Special cases

In the absence of nanoparticles, we obtained the solution of Fetecau et al.^33^.

38 $$\theta \left( {\varepsilon ,t} \right) = \frac{2\sqrt t }{{\sqrt {\Pr_{eff} } }}\left[ {\frac{1}{\sqrt \pi }e^{{ - \varepsilon \frac{{\Pr_{eff} }}{4t}}} - \varepsilon \frac{{\sqrt {\Pr_{eff} } }}{2\sqrt t }erfc\left( {\varepsilon \frac{{\sqrt {\Pr_{eff} } }}{2\sqrt t }} \right)} \right].$$

When we put $K = 0{\text{ and }}Sc = 1$ in Eq. (11), we get the solution in the form of as given under:

39 $$C(\varepsilon ,t) = \sqrt {\frac{4t}{\pi }} e^{{ - \frac{{\varepsilon^{2} }}{4t}}} - \varepsilon erfc\left( {\frac{\varepsilon }{2\sqrt t }} \right)$$

In the absence of Maxwell fluid coefficient (Newtonian fluid $\lambda = 0$) in Eq. (9), we acquired the following solution:

40 $$\begin{gathered} u(\varepsilon ,t) = \int\limits_{0}^{t} {e^{i\omega (t - v)} W_{13} (\varepsilon ,v)dv - \frac{{a_{2} Gr_{t} }}{{\left( {a_{0} \Pr_{eff} - a_{1} } \right)\sqrt {a_{0} \Pr_{eff} } }}\left( {\frac{{e^{{ - \frac{{\varepsilon^{2} }}{4t}}} \sqrt t \left( {4t + \varepsilon^{2} } \right)}}{3\sqrt \pi } - \frac{1}{6}\left( {6t\varepsilon + \varepsilon^{3} } \right)erfc\left( {\frac{\varepsilon }{2\sqrt t }} \right)} \right)} \hfill \\ \, + \frac{{a_{3} Gr_{m} }}{{\sqrt {Sc} }}\int\limits_{0}^{t} {\frac{{e^{{ - \frac{{m_{1} }}{{m_{2} }}(t - v)}} }}{{m_{2} }}} W_{14} \left( {\varepsilon ,v} \right)dv + \frac{{a_{2} Gr_{t} }}{{\left( {a_{0} \Pr_{eff} - a_{1} } \right)\sqrt {a_{0} \Pr_{eff} } }}\left( {\frac{{e^{{ - \frac{{a_{0} \Pr_{eff} \varepsilon^{2} }}{4t}}} \sqrt t \left( {4t + a_{0} \Pr_{eff} \varepsilon^{2} } \right)}}{3\sqrt \pi }} \right) \hfill \\ \, - \frac{{a_{2} Gr_{t} }}{{\left( {a_{0} \Pr_{eff} - a_{1} } \right)\sqrt {a_{0} \Pr_{eff} } }}\left( {\frac{1}{6}\left( {6t\sqrt {a_{0} \Pr_{eff} } \varepsilon + \left( {\sqrt {a_{0} \Pr_{eff} } \varepsilon } \right)^{3} } \right)erfc\left( {\frac{{\sqrt {a_{0} \Pr_{eff} } \varepsilon }}{2\sqrt t }} \right)} \right) - \frac{{a_{3} Gr_{m} }}{{\sqrt {Sc} }}\int\limits_{0}^{t} {\frac{{e^{{ - \frac{{m_{1} }}{{m_{2} }}(t - v)}} }}{{m_{2} }}} W_{6} \left( {\varepsilon ,v} \right)dv \hfill \\ \end{gathered}$$

where

$$W_{13} (\varepsilon ,t) = \left\{ {\frac{{e^{{ - \frac{{\varepsilon^{2} }}{4t}}} \varepsilon }}{{2\sqrt \pi t^{\frac{3}{2}} }}, \, \varepsilon > 0} \right., \, W_{14} (\varepsilon ,t) = \frac{{ie^{ - Kt} }}{2}\left[ {e^{i\varepsilon \sqrt K } erfc\left\{ {\frac{\varepsilon }{2\sqrt t } + i\sqrt {Kt} } \right\} - e^{ - i\varepsilon \sqrt K } erfc\left\{ {\frac{\varepsilon }{2\sqrt t } - i\sqrt {Kt} } \right\}} \right]$$

## Numerical results and discussion

In order to see the physical meaning of the problem, we use the LT method to obtain the solution for temperature, concentration, velocity, rate of heat transfer and rate of mass transfer. These solutions have been studied graphically by giving numerical values to various embedded parameters like radiation factor, chemical reaction factor, thermal Grashof number, mass Grashof number, Maxwell fluid coefficient, Schmidt number, Prandtl number. The value of volume fraction parameter is taken 0.01.

**Figure 2:**
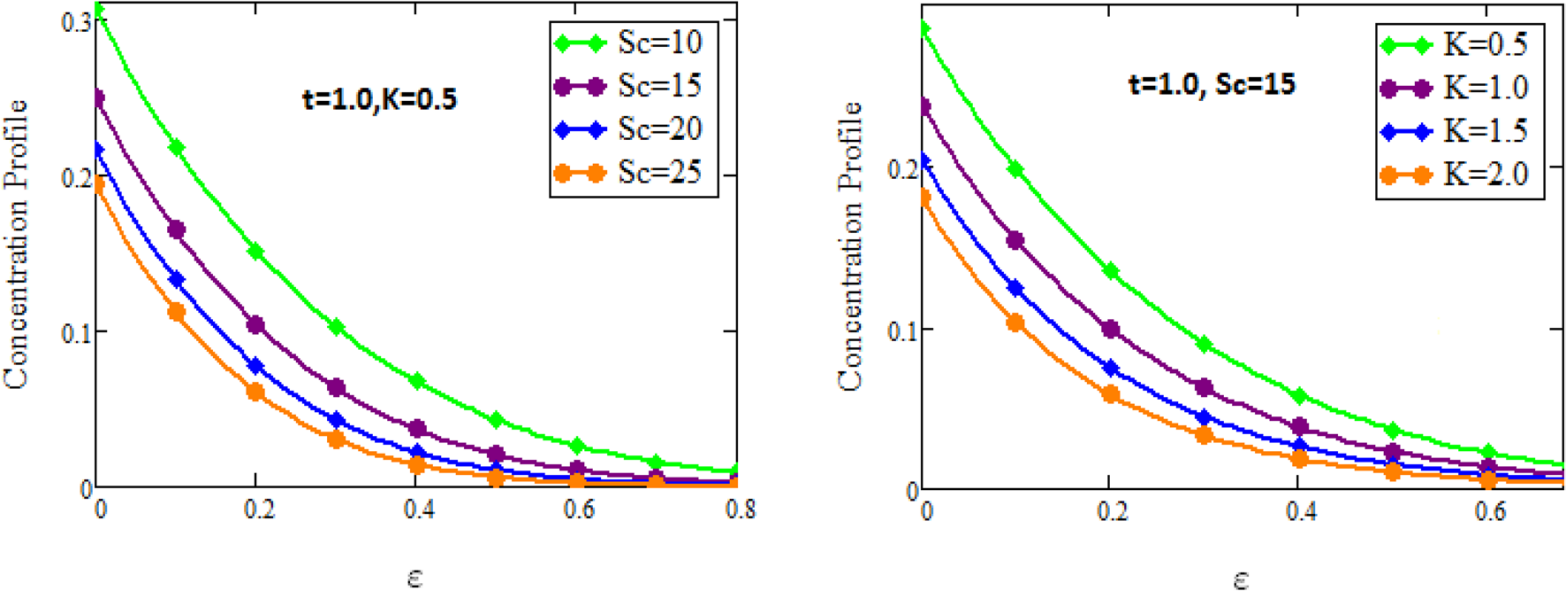
Concentration curves for various values of *Sc* and *K.*

**Figure 3:**
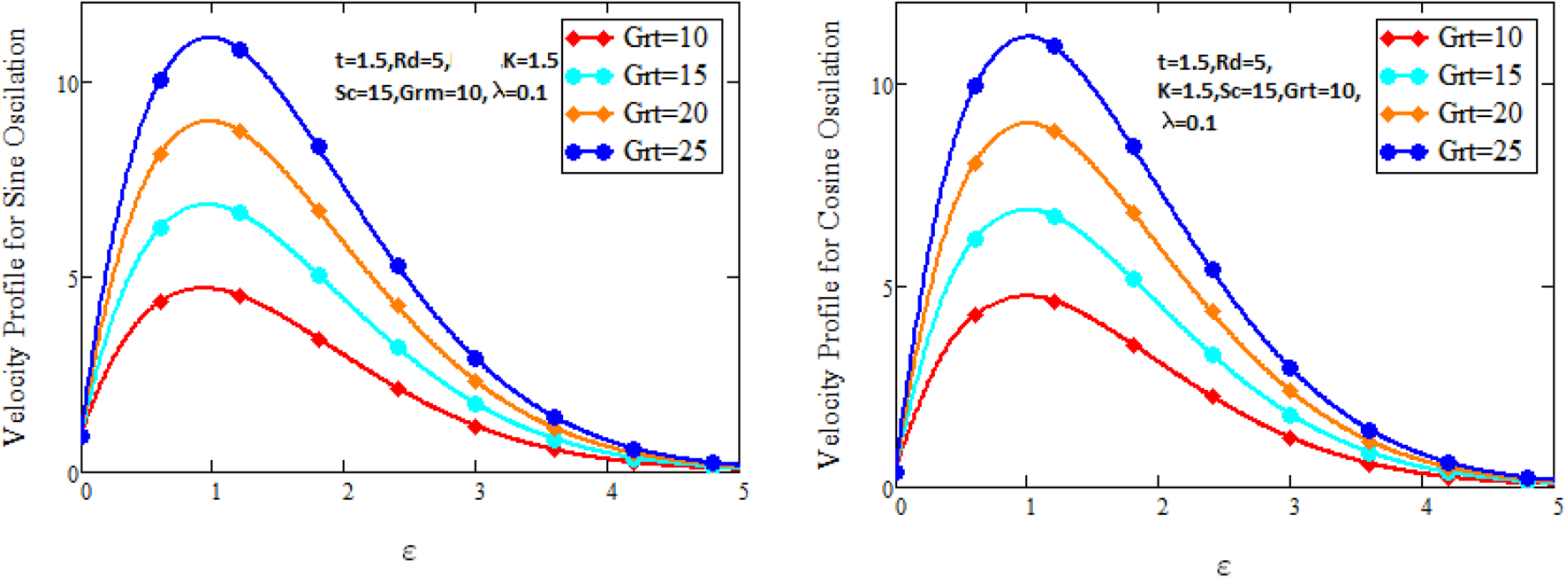
Velocity curves for various values of $Gr_{t}$.

**Figure 4:**
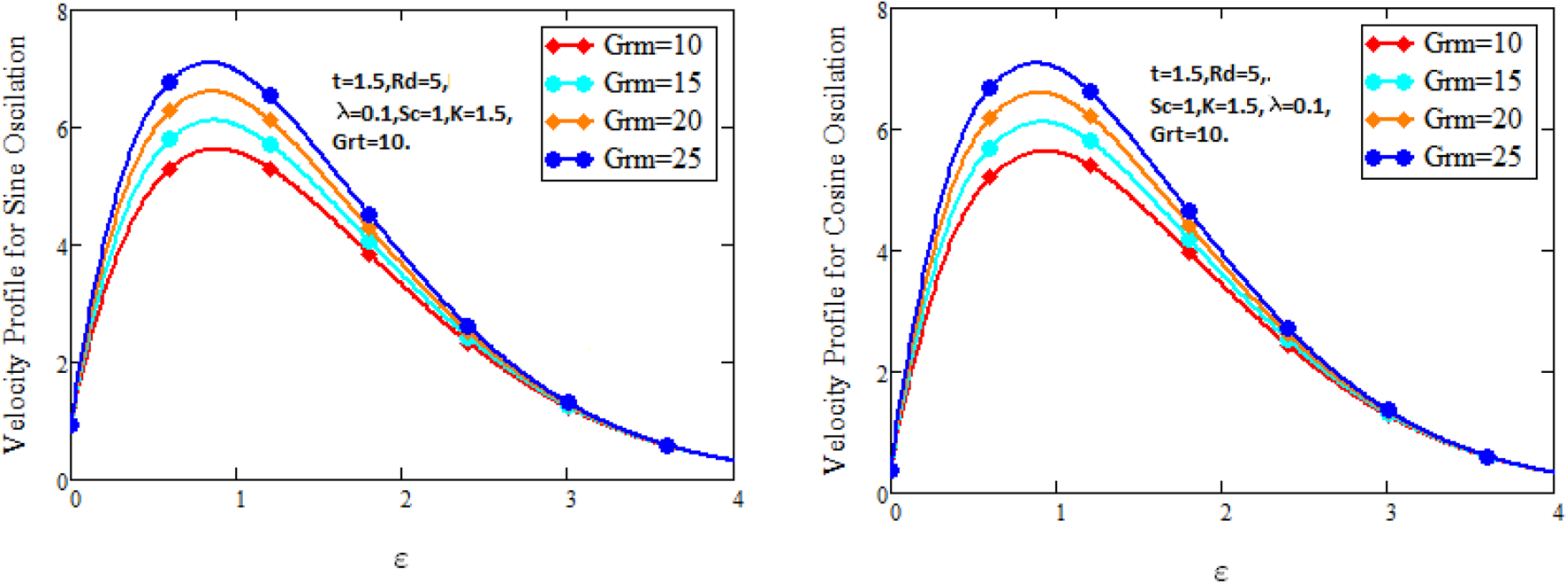
Velocity curves for various values of $Gr_{m} .$

**Figure 5:**
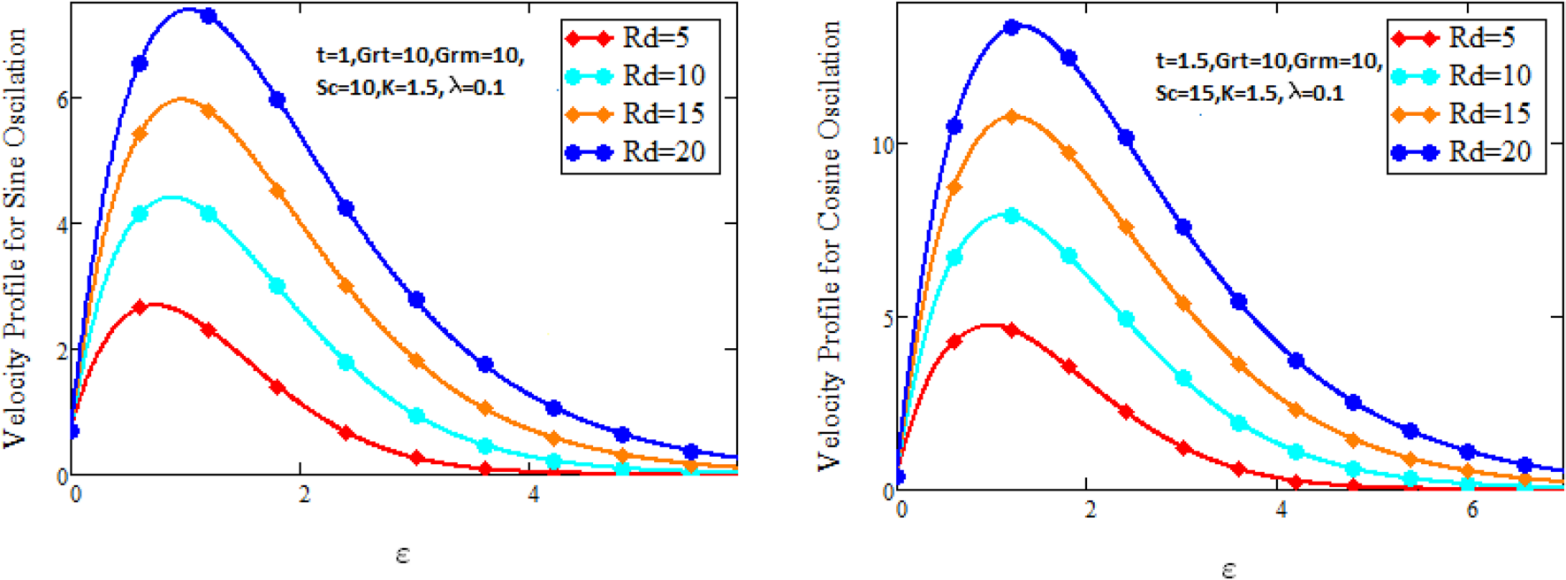
Velocity curves for various values of $Rd.$

**Figure 6:**
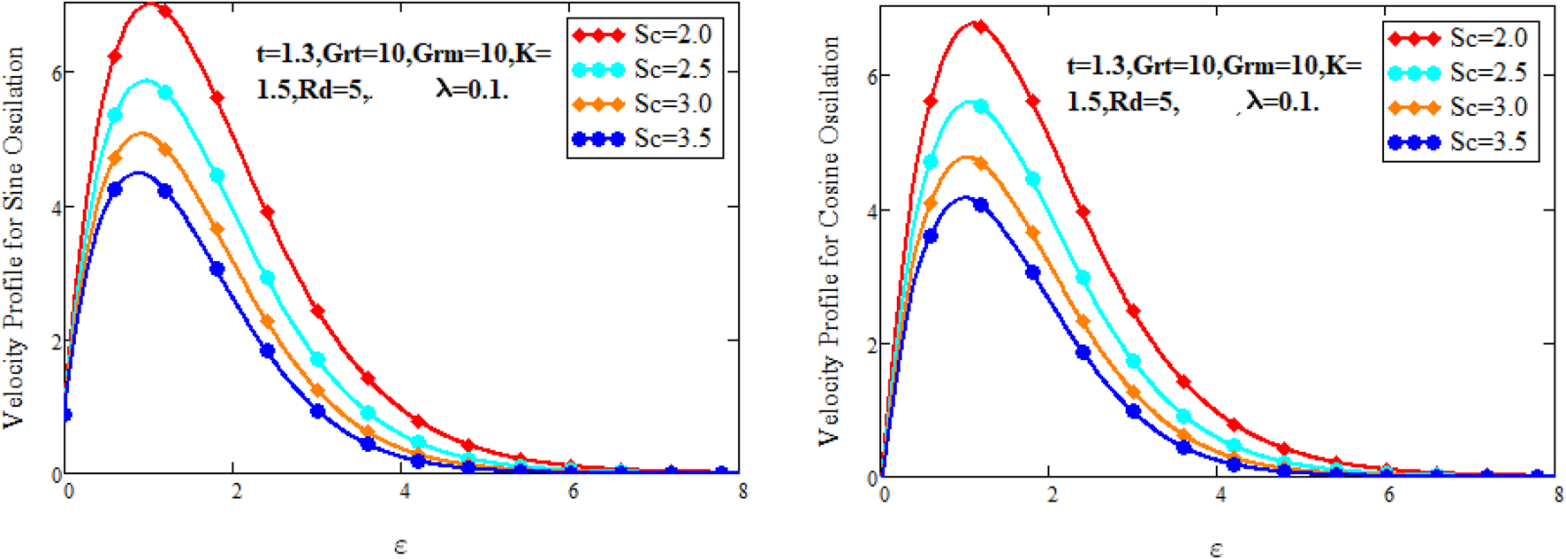
Velocity curves for various values of $Sc.$

**Figure 7:**
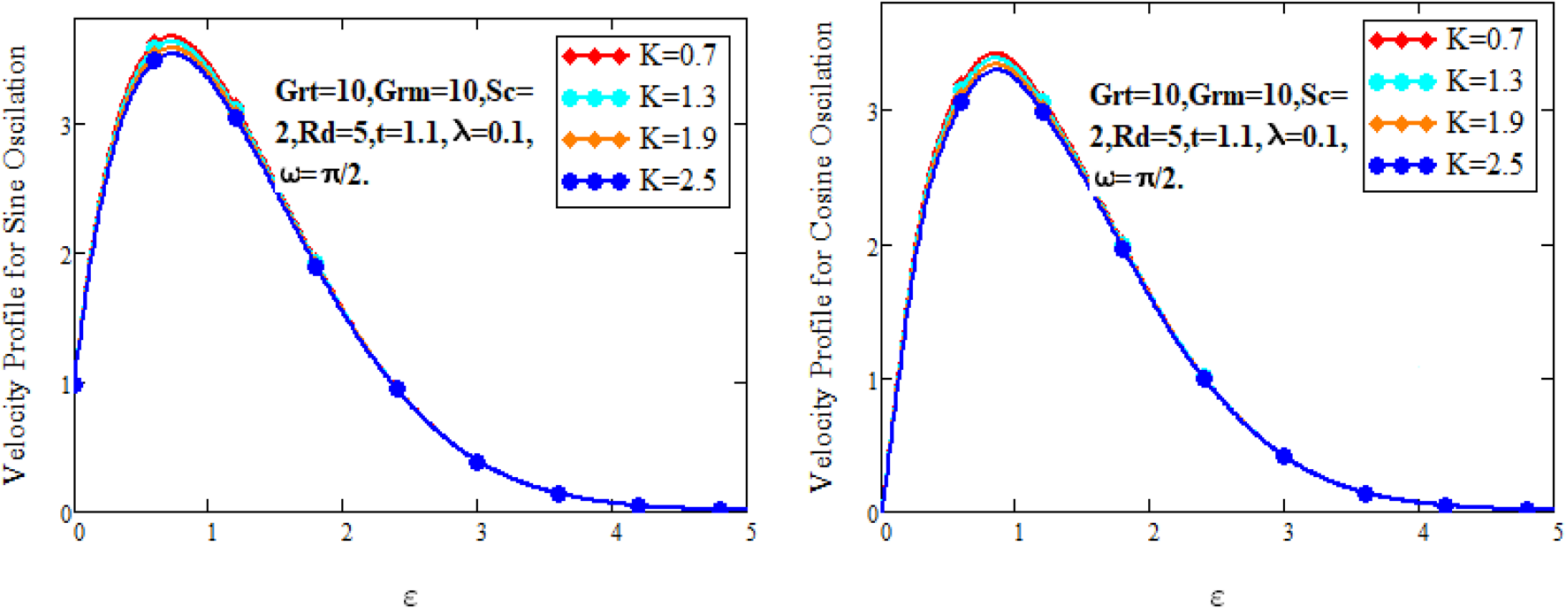
Velocity curves for various values of $K$.

**Figure 8:**
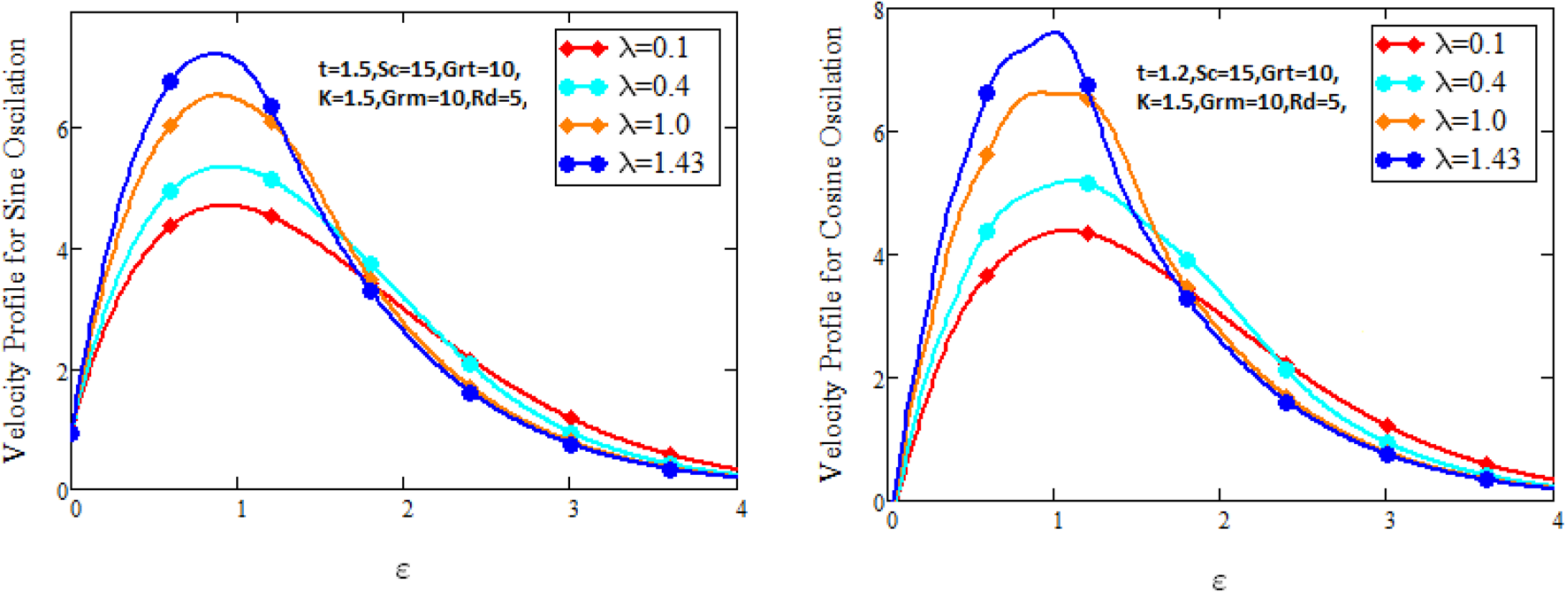
Velocity curves for various values of $\lambda$.

Figure 2 characterizes the concentration for variations of Schmidt number $Sc$ and chemical reaction factor $K.$ It is found that by increasing the value of Schmidt number $Sc$ and chemical reaction factor $K$, the concentration of the nanofluid decreases. Physically, there is inverse relation between Schmidt number and mass diffusivity. As we enhance Schmidt number $Sc$, the mass diffusion is de-escalates. Thus, concentration profile decreases. Similarly, concentration profile decreases with the increasing estimation of chemical reaction factor $K$. This behavior is due to less fluid particles are produced as a product. In Fig. 3 the flow profile of Maxwell fluid is studied under the revamping of thermal Grashof number for both the sine and cosine oscillations. The velocity distribution for both sine and cosine oscillation is the growing function as we grow the value of thermal Grashof number $Gr_{t}$. Physically, this characteristic is because of the viscous and thermal buoyancy forces in flow of fluid. The greater the value of $Gr_{t}$ shows the fluid is heated that bolsters the impact of thermal buoyancy forces because of the existence of convection currents. These currents get the value of great importance due to prevailing temperature slop and eventually cause the viscous forces to sink. As a result, the fluid’s velocity enhances. Figure 4 displays the impact of mass Grashof number on velocity. It is also have same behavior like Fig. 3 i.e. the enhancement of $Gr_{m}$ enhances the velocity of the fluid. This is due to the enhancement in mass buoyancy force and buoyancy force enhances concentration gradient, which result enhances the velocity. Figure 5 portrays the behavior of radiation coefficient $Rd$ for both sine and cosine oscillation. It characterize that the fluid’s velocity accelerated with the greater value of $Rd$. Physically, rate of energy transfer explains this increase. As $Rd$ increments, rate of energy transfer to the fluids grows which results to weak the bond between fluid particles. As a result these poorly associated particles collectively give much weaker viscosity to fluid motion and gradually fluid gets accelerated. Figure 6 shows the relationship between Schmidt number and velocity of the fluid. It is spotted that the increases in Schmidt number decelerate the fluid’s velocity for both the oscillations. Physically, as Schmidt number $Sc$ increases, the molecular diffusivity reduces due to which velocity decreases. Figure 7 exhibits the effects of chemical reaction factor on velocity distribution for both the sine and cosine oscillation. Clearly Fig. 7 demonstrates the de-escalation in fluid velocity as we grow the value of chemical reaction factor. The variation of velocity distribution because of Maxwell fluid coefficient $\lambda$ for both the oscillation is described in Fig. 8. It is realized the fluid flow is increasing function for greater value of $\lambda .$ Physically, this observation is because of the retard in boundary layer thickness. Velocity shows the significant behavior in the main stream region and finally approaching to zero.

**Figure 9:**
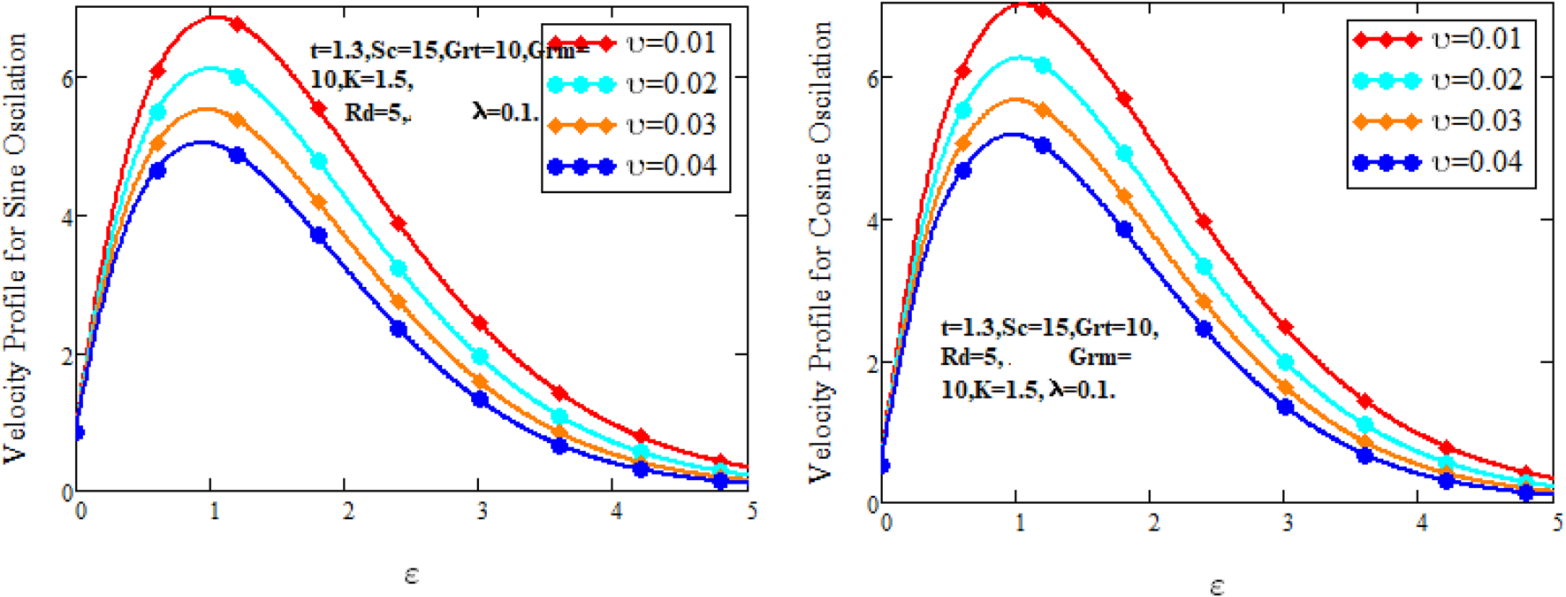
Velocity curves for various values of $\vartheta$.

Figure 9 shows the behavior of graphene nanoparticle on velocity profile. It can be seen that the velocity of nanofluid reduces for the growing value of volume fraction. This is because of increases the nanoparticles makes denser the fluid, so its velocity decelerates.

**Figure 10:**
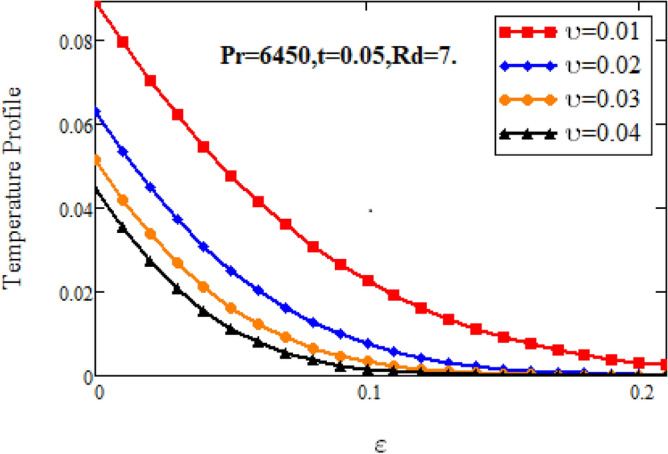
Temperature profiles for various values of $\vartheta$.

**Figure 11:**
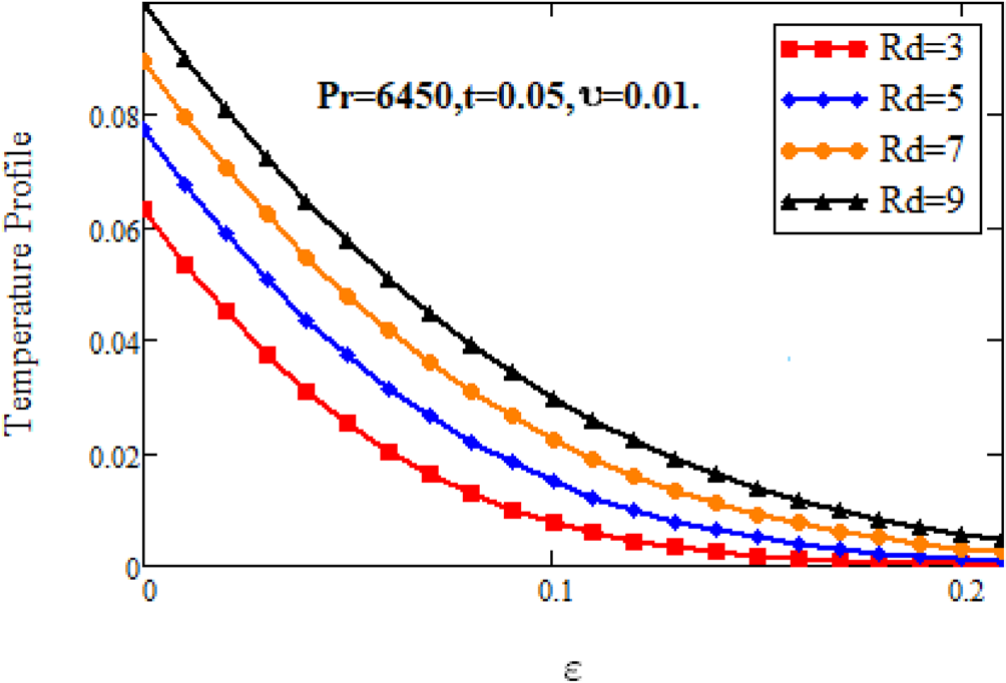
Temperature profiles for various values of *Rd.*

Figures 10 and 11 highlight the impact of various parameters on temperature profile of nano fluid. Figure 10 depicts the influence of volume fraction on temperature profile of nanofluid. It is observed that the temperature of nanofluid decelerates with accelerating the estimations of volume factor. Physically, this behavior is due to decrease of thermal conductivity on adding nanoparticles, which results decelerates the temperature of nanofluid. Figure 11 shows the increase in nanofluid’s temperature with increasing radiation parameter. Since increase in at fixed value of and, decelerates the value of, therefore slop of radioactive heat flux increases which lead to grow the radiative heat transfer rate and gradually the fluid’s temperature increments, It means that thickness of energy boundary layer reduces and temperature is distributed more uniformly.

**Figure 12:**
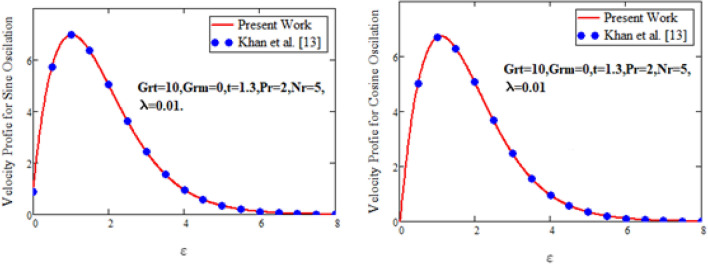
Comparative study of velocity profiles.

**Figure 13:**
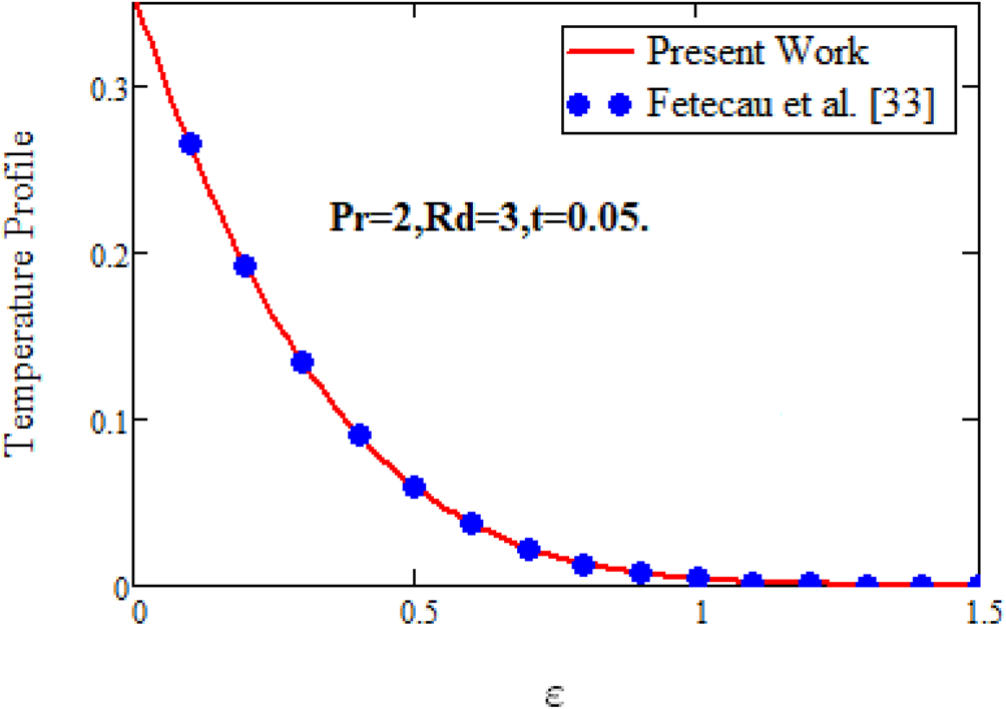
Comparative study of temperature profiles.

In order to authenticate our present solutions, Figs. 12 and 13**,** are presented. It can be observed if the volume fraction parameter $\vartheta$ are removed from temperature field and $\vartheta {\text{ and }}Gr_{m}$ are deleting from the velocity field of the current model, then the present solutions for temperature and velocity field are in excellent agreement with the velocity solution of ordinary Maxwell fluid model of Khan et al.^13^ for both sine and cosine oscillations and the temperature solution of Fetecau et al.^33^.

## Conclusion

The aspiration of this work was to evaluate the oscillating Maxwell nanofluid with heat and mass transfer. The analytical solution for temperature, concentration, and velocity were obtained through LT method. The rate of heat and mass transfer was also measured in the form of Nusslet number and Sherwood number. Finally, the effect of different physical factors were shown in discussion section graphically and theoretically for both sine and cosine oscillation. The solution of Newtonian fluid was also analyzed as a special case. Following are the key concepts of this work (Supplementary Information S1):

- The concentration profile is decreasing function for Schmidt number *Sc*.
- Decrease occurs in concentration with increasing the estimation of chemical reaction factor $K.$
- The velocity of the nanofluid grows, when thermal Grashof number $Gr_{t}$ is accelerated.
- The nanofluid’s velocity enhances with the enhancement of mass Grashof number $Gr_{m}$.
- The velocity field is accelerated as we accelerate the estimation of Maxwell fluid parameter $\lambda .$
- Reduction occurs in velocity with higher value of Schmidt number $Sc.$
- The nanofluid’s velocity is increasing function as we increase the value of radiation parameter $Rd$ while decreasing function against the chemical reaction factor $K.$
- Volume function also reduces the nanofluid’s velocity.
- The temperature of fluid is growing function when radiation parameter $Rd$ is increases, while falls when volume fraction parameter $\vartheta$ enlarges.
- Nusslet number and Sherwood number are constant.

## Supplementary Information

Supplementary Information.

## Data Availability

All data generated or analyzed during this study are included in this article.

## Abbreviations

$$T_{\infty }$$: Ambient temperature (K)
*g*: Gravitational acceleration (ms^−2^)
$\beta_{m}$: Coefficient of thermal expansion (m^3^/kg)
$\lambda$: Maxwell fluid parameter (s)
*k*: Thermal conductivity (Wm^−1^ K^−1^)
*ht*: Heat transfer coefficient (Wm^−2^ K^−1^)
$$\varepsilon$$: Space variable (m)
*u*: Velocity of fluid (ms^−1^)
*Gr*_*m*_: Mass Grashof Number
*r*: Laplace parameter
*C*: Dimensionless concentration
*nf*: Nanofluid
$$\mu_{nf}$$: Dynamic viscosity
$q_{r}$: Radiative heat flux
$C_{\infty }$: Ambient concentration (kg/m^3^)
$\beta_{t}$: Coefficient of thermal expansion (K^−1^)
$\rho$: Fluid density (kg m^3^)
$$\upsilon$$: Kinematic viscosity (m^2^ s^−1^)
$c_{p}$: Specific heat (J kg^−1^ K^−1^)
*t*: Time (s)
$$\theta$$: Dimensionless temperature
$Gr_{t}$: Thermal Grashof number
$$Rd$$: Radiation parameter
Pr: Prantl number
LT: Laplace transform
*K*: Chemical reaction parameter
*hc*: Mass transfer coefficient
*M*: Diffusion specie coefficient
