# Author Correction: Closed-form solution of oscillating Maxwell nano-fluid with heat and mass transfer

**DOI:** 10.1038/s41598-022-20901-5

**Published:** 2022-09-28

**Authors:** Aamir Farooq, Sadique Rehman, Abdulaziz N. Alharbi, Muhammad Kamran, Thongchai Botmart, Ilyas Khan

**Affiliations:** 1 Department of Mathematics, Zhejiang Normal University, Jinhua, 321004 Zhejiang China; 2 Department of Mathematics, Abbottabad University of Science and Technology, Abbottabad, Pakistan; 3 Department of Pure and Applied Mathematics, University of Haripur, Haripur, KPK Pakistan; 4 Department of Physics, College of Science, Taif University, P.O.Box 11099, Taif, 21944 Saudi Arabia; 5 Department of Mathematics, COMSATS University Islamabad, Wah Campus, Islamabad, 47040 Pakistan; 6 Department of Mathematics, Faculty of Science, Khon Kaen University, Khon Kaen, 40002 Thailand; 7 Department of Mathematics, College of Science Al‑Zulfi, Majmaah University, Al‑Majmaah, 11952 Saudi Arabia

Correction to: *Scientific Reports* 10.1038/s41598-022-16503-w, published online 16 July 2022

The Acknowledgments section in the original version of this Article was omitted. The Acknowledgments section now reads:

“Abdulaziz N. Alharbi would like to acknowledge the financial support of Taif University Researchers Supporting Project number (TURSP-2020/319), Taif University, Taif, Saudi Arabia".

The original Article has been corrected.

