# Supplementary material for: Closed-form solution of oscillating Maxwell nano-fluid with heat and mass transfer
Source: Sci Rep. 2022 Jul 16;12:12205. doi: 10.1038/s41598-022-16503-w (PMC9288494; doi:10.1038/s41598-022-16503-w)
Supplement: Supplementary file 1 — Supplementary Information. [file 41598_2022_16503_MOESM1_ESM.docx]

1. **Appendix A**$\begin{matrix} \Pr_{eff}=\frac{\Pr}{1+Rd},b_{0}=\frac{1}{\lambda},b_{1}=\frac{a_{1}-a_{0}\Pr_{eff}}{\lambda},b_{11}=\frac{a_{0}\Pr_{eff}-a_{1}}{\lambda},b_{2}=\frac{ScK}{\lambda},b_{3}=\frac{Sc}{\lambda},b_{4}=b_{0}-b_{3} \\ m=a_{0}\Pr_{eff}-1,m_{1}=ScK,m_{2}=Sc-a_{1},a_{0}=\frac{\vartheta_{5}}{\vartheta_{6}},a_{1}=\frac{\vartheta_{1}}{\vartheta_{2}},a_{2}=\frac{\vartheta_{3}}{\vartheta_{2}},a_{3}=\frac{\vartheta_{4}}{\vartheta_{2}}. \end{matrix}$
